# Sizing the Problem of Improving Discovery and Access to NIH-Funded Data: A Preliminary Study

**DOI:** 10.1371/journal.pone.0132735

**Published:** 2015-07-24

**Authors:** Kevin B. Read, Jerry R. Sheehan, Michael F. Huerta, Lou S. Knecht, James G. Mork, Betsy L. Humphreys, NIH Big Data Annotator Group

**Affiliations:** 1 Medical Library, NYU Langone Medical Center, New York, New York, United States of America; 2 National Library of Medicine, National Institutes of Health, Bethesda, Maryland, United States of America; 3 National Institutes of Health, Bethesda, Maryland, United States of America; Université de Montréal, CANADA

## Abstract

**Objective:**

This study informs efforts to improve the discoverability of and access to biomedical datasets by providing a preliminary estimate of the number and type of datasets generated annually by research funded by the U.S. National Institutes of Health (NIH). It focuses on those datasets that are “invisible” or not deposited in a known repository.

**Methods:**

We analyzed NIH-funded journal articles that were published in 2011, cited in PubMed and deposited in PubMed Central (PMC) to identify those that indicate data were submitted to a known repository. After excluding those articles, we analyzed a random sample of the remaining articles to estimate how many and what types of invisible datasets were used in each article.

**Results:**

About 12% of the articles explicitly mention deposition of datasets in recognized repositories, leaving 88% that are invisible datasets. Among articles with invisible datasets, we found an average of 2.9 to 3.4 datasets, suggesting there were approximately 200,000 to 235,000 invisible datasets generated from NIH-funded research published in 2011. Approximately 87% of the invisible datasets consist of data newly collected for the research reported; 13% reflect reuse of existing data. More than 50% of the datasets were derived from live human or non-human animal subjects.

**Conclusion:**

In addition to providing a rough estimate of the total number of datasets produced per year by NIH-funded researchers, this study identifies additional issues that must be addressed to improve the discoverability of and access to biomedical research data: the definition of a “dataset,” determination of which (if any) data are valuable for archiving and preservation, and better methods for estimating the number of datasets of interest. Lack of consensus amongst annotators about the number of datasets in a given article reinforces the need for a principled way of thinking about how to identify and characterize biomedical datasets.

## Introduction

Biomedical research is becoming increasingly data-centric. The proliferation of low-cost methods for whole genome sequencing, growing use of functional magnetic resonance imaging (fMRI) and other imaging modalities, and more widespread availability of clinical data in electronic health records (EHRs) are among the factors enabling biomedical researchers to generate and make use of increasing volumes of digital data in their research. Growth in the availability of biomedical data is, in turn, generating growing interest in improving the management and utilization of the many types of data (e.g., genomic, imaging, behavioral, clinical, exposure) that are used in biomedical research.

Improved management of biomedical research data—or any scientific data—can have many benefits. Fundamentally, improved management of scientific data is essential to the preservation of the scientific record, of which data are a growing part. It is also the basis for improved sharing of data, for example, enabling other researchers to have access to previously collected data. Data sharing can improve the quality and efficiency of research by allowing researchers to verify and validate prior research findings, to conduct research that combines previously collected data with newly collected data, and to compare the results of related research studies more easily [1, 2].

Around the world, governments, research funding organizations, and investigators are actively pursuing better management of, and access to scientific research data [3]. The European Commission, European Research Council and Canadian Institute of Health Research have all established policies for research data [4–6]. In the United States, a February 2013 memorandum from the White House Office of Science and Technology Policy directed all U.S. federal science agencies that spend more than $100 million per year on research and development to develop plans to increase public access to digital data resulting from research funded by those agencies [7]. The U.S. Department of Health and Human Services issued its plans in February 2015 [8].

The U.S. National Institutes of Health (NIH), part of the Department of Health and Human Services, is the world’s largest funder of biomedical research. It invests approximately $30 billion per year in biomedical research, most of which is expended through competitive grants to more than 300,000 researchers at universities, medical schools, and other research institutions in every U.S. state and around the world [9]. The NIH Big Data to Knowledge (BD2K) initiative launched in 2013 aims to “enable biomedical scientists to capitalize more fully on the Big Data being generated by those research communities” [10]. One goal of BD2K is to develop effective and efficient mechanisms to enable the identification of, access to, and citation for biomedical data, bringing more data into the ecosystem of science and scholarship [11].

An important step in designing, developing, and implementing mechanisms to discover, access, and cite the biomedical data used in NIH-funded research is to characterize the number of new datasets generated annually by NIH-funded researchers, the types of data created, and the frequency of reuse of existing data. Of particular interest are “invisible” datasets—datasets that are not currently stored and made accessible via well-known, publicly accessible data repositories. Previous studies have estimated how much data is shared by analyzing a set number of journals [12, 13], performed analyses on how often specific datasets were cited in the literature [14, 15], and used complex algorithms to estimate the entire universe of data for a specific discipline [16]. While it has been shown that it is possible to make some estimates of the types of data that are currently deposited in known repositories, it is more challenging to estimate the number of datasets that are *not* publicly or systematically registered, deposited, or archived. Arguably, such datasets should be a primary focus of any effort to improve the discoverability and reuse of data because they are less discoverable and accessible than data deposited in a known repository.

We conducted a study to develop a preliminary estimate of the annual volume and types of datasets generated by NIH-funded researchers. This study was undertaken to inform initial NIH efforts to improve the discoverability of and access to biomedical datasets. For the purpose of this study, a dataset was defined as any collection of data (e.g., different type of measurement) that was generated or reused to inform the results described in an article.

## Methods

**Fig 1:**
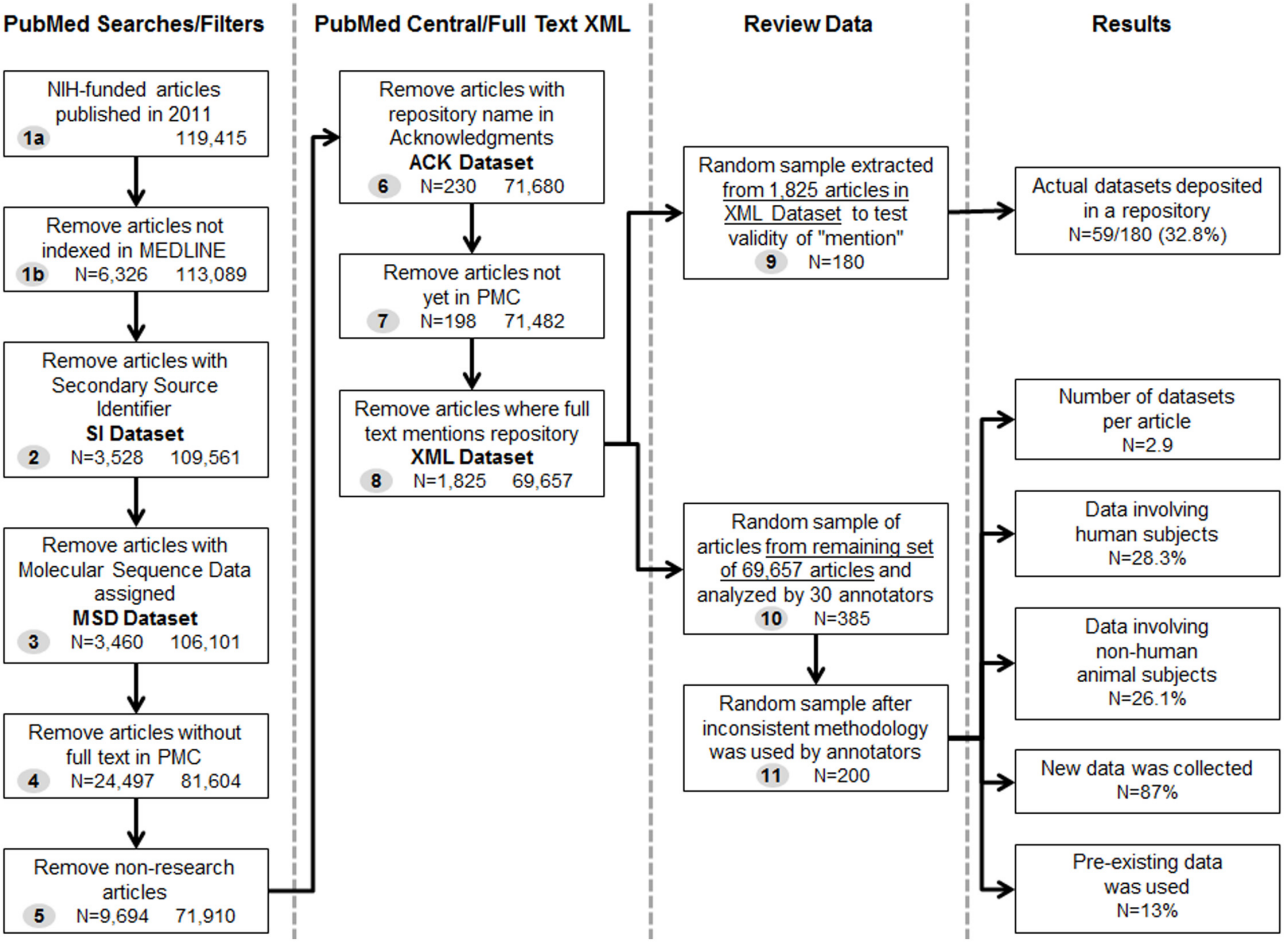
Diagram of process taken to identify NIH-funded datasets via the published journal literature (Including Results).

Our approach to characterizing biomedical research datasets relied on an examination of datasets that are used or generated in the course of research that is reported in published journal literature. This approach misses datasets that are collected as part of a research project but are not reported in a publication. While little is known about the full extent of non-publication in biomedical research, recent work indicates that as long as four years after study completion, the results from approximately one-third of clinical trials registered in ClinicalTrials.gov remains unpublished [17]. There is also evidence that the availability and discoverability of research datasets declines rapidly with age [18]. To the extent that discovery of datasets may be enabled by linking data to associated journal articles, our approach was a reasonable first step toward quantification and characterization. We further restrict our analysis to datasets generated by NIH-funded research. While this does not represent all of biomedical research, it is research that is subject to U.S. policies that require expanded data sharing. This sample also covers a broad spectrum of biomedical research types, from basic to clinical research across a wide range of diseases, conditions, and systems and therefore was a good starting point for analysis. For clarity, the process taken to identify NIH-funded datasets via the published journal literature described below is also illustrated in Fig 1.

### Identifying articles with datasets deposited in known repositories

To estimate the number of datasets generated annually from NIH-funded research and identify datasets that are not stored in a known repository, our analysis focused on NIH-funded articles that were published in 2011. These articles represented the most current complete set of articles for a given year at the time of our study. To retrieve these articles, we searched PubMed using the strategy illustrated below (Table 1-a), which retrieved citations from articles published in 2011 that acknowledged research funding support from NIH (step 1a, Fig 1). Use of PubMed’s Publication Type [PT] and Grant [GR] search tags enabled us to focus the search on citations that received NIH funding. This search identified almost 120,000 citations. We further limited the search to include those citations indexed in MEDLINE (using the MEDLINE [sb] subset search tag). This step focuses the analysis on citations that have been fully indexed and contain additional information to indicate whether datasets used in the summarized research were deposited in a known data repository. This search (Table 1-b) retrieved more than 113,000 articles. All bolded text in Tables 1–5 indicate search terms that were progressively added to the search string (step 1b, Fig 1).

**Table 1:** PubMed searches identifying articles with funding support from the NIH.

a)	2011 [dp] AND (NIH [gr] OR Research Support, N.I.H., Extramural [pt] OR Research Support, N.I.H., Intramural [pt])	119,415
b)	2011 [dp] AND (NIH [gr] OR Research Support, N.I.H., Extramural [pt] OR Research Support, N.I.H., Intramural [pt]) **AND medline [sb]**	113,089

**Table 2:** PubMed searches identifying when datasets were deposited in certain repositories (SI dataset).

2011 [dp] AND (NIH [gr] OR Research Support, N.I.H., Extramural [pt] OR Research Support, N.I.H., Intramural [pt]) AND medline [sb] **AND (GDB [si] OR GENBANK [si] OR OMIM [si] OR PDB [si] OR PIR [si] OR RefSeq [si] OR SWISSPROT [si] OR ClinicalTrials.gov [si] OR ISRCTN [si] OR GEO [si] OR PubChem-Substance [si] OR PubChem-Compound [si] OR PubChem-BioAssay [si])**	3528

**Table 3:** PubMed search identifying articles with the “Molecular Sequence Data” MeSH Heading (MSD dataset).

2011 [dp] AND (NIH [gr] OR Research Support, N.I.H., Extramural [pt] OR Research Support, N.I.H., Intramural [pt]) AND medline [sb] **NOT** (GDB [si] OR GENBANK [si] OR OMIM [si] OR PDB [si] OR PIR [si] OR RefSeq [si] OR SWISSPROT [si] OR ClinicalTrials.gov [si] OR ISRCTN [si] OR GEO [si] OR PubChem-Substance [si] OR PubChem-Compound [si] OR PubChem-BioAssay [si]) **AND molecular sequence data [mh:noexp]**	3460

**Table 4:** PubMed search identifying articles in PMC.

2011 [dp] AND (NIH [gr] OR Research Support, N.I.H., Extramural [pt] OR Research Support, N.I.H., Intramural [pt]) AND medline [sb] **NOT** (GDB [si] OR GENBANK [si] OR OMIM [si] OR PDB [si] OR PIR [si] OR RefSeq [si] OR SWISSPROT [si] OR ClinicalTrials.gov [si] OR ISRCTN [si] OR GEO [si] OR PubChem-Substance [si] OR PubChem-Compound [si] OR PubChem-BioAssay [si]) **NOT** molecular sequence data [mh:noexp] **AND pubmed pmc all[sb]**	81604

**Table 5:** Removal of articles that were not considered “research”.

2011 [dp] AND (NIH [gr] OR Research Support, N.I.H., Extramural [pt] OR Research Support, N.I.H., Intramural [pt]) AND medline [sb] NOT (GDB [si] OR GENBANK [si] OR OMIM [si] OR PDB [si] OR PIR [si] OR RefSeq [si] OR SWISSPROT [si] OR ClinicalTrials.gov [si] OR ISRCTN [si] OR GEO [si] OR PubChem-Substance [si] OR PubChem-Compound [si] OR PubChem-BioAssay [si]) NOT molecular sequence data [mh:noexp] AND pubmed pmc all[sb] **NOT review [pt] NOT letter [pt] NOT news [pt] NOT editorial [pt]**	71910

From this set of articles, we identified those that indicated when authors shared their data in a specific repository. This process began by searching for articles that had a Secondary Source Identifier [SI][19]; this identifier indicates when the author of an article has deposited his/her data in one of the specific repositories that are recognized in MEDLINE/PubMed. The repositories that can be designated in the SI field include, but are not limited to: ClinicalTrials.gov, PubChem, Johns Hopkins University Genome Data Bank, Gene Expression Omnibus, GenBank, ISRCTN Register, Mendelian Inheritance in Man, Protein Data Bank, Protein Identification Resource, Reference Sequence, and SWISSPROT Protein Sequence Database. The aforementioned repositories provide evidence of how many articles acknowledge data deposition in each of these locations within a given year (Table 2). This step identified 3,528 (**SI dataset**) articles that had deposited data into one of the listed repositories (step 2, Fig 1).

We removed the **SI dataset** articles from our article set and searched the remaining articles for the Medical Subject Heading (MeSH) “Molecular Sequence Data.” Citations tagged with this MeSH heading are those for which data are likely to be deposited in GenBank or an equivalent repository. For the year 2011 this search identified 3,460 (**MSD dataset**) articles that provided indication that data should have been deposited in a repository (Table 3; step 3, Fig 1).

We removed the **MSD dataset** articles from the sample and searched the remaining articles for those with full-text available in PubMed Central (PMC) using the [sb] search tag. This allowed us to conduct further analysis on information that would be provided only in the full-text of an article (as opposed to the MEDLINE citation) (Table 4; step 4, Fig 1).

We reduced the set of articles by identifying and removing all non-research articles, meaning those with the MEDLINE publication type [PT] of review, editorial, news, and letter. This step created a sample that included only full-text research articles with MEDLINE records that did not mention depositing data into a repository (Table 5). This process resulted in a sample of 71,910 articles (step 5, Fig 1).

**Fig 2:**
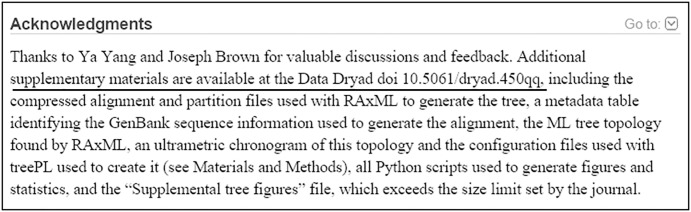
Example of the PubMed Central Acknowledgments where the authors have indicated the deposit of data in a specific repository; PMCID: PMC4085032.

We then examined the articles to identify those that mention the sharing of their data in the acknowledgments section of an article, using the Acknowledgements search field [20] of PMC (Fig 2) [21]. The Acknowledgments section of a full-text article is often used to indicate when data have been shared in a specific repository. We selected the NIH Data Sharing Repositories Web page [22] as our gold standard to gather a list of NIH-specific data repositories, and used keyword variations and acronyms (e.g., Gene Expression Omnibus, GEO, Protein Data Bank, PDB) to search each repository in the Acknowledgments field in PMC with the [ack] search tag for the year 2011. Additionally, the terms “DataCite” and “Dryad” were added to the strategy, seeking occurrences in any PMC search field, because they are well-known resources for discovery of scientific data, including data referenced in scientific journal articles.

This search identified 814 (**ACK dataset**) articles that mentioned one or more of the recognized repositories. After accounting for overlap with the **SI dataset** and **MSD dataset**, we removed 230 articles in the **ACK dataset** from our sample set, leaving us with 71,680 articles that made no mention that their data were deposited in a known repository (step 6, Fig 1). Of these articles, 198 were not yet available in PMC at the time of our study, so they were removed from the sample, leaving 71,482 articles (step 7, Fig 1).

The final procedure used to identify articles that mention the deposit of data was to scan for the same keyword variations and acronyms from the 45 NIH data repositories within the XML full-text data for the remaining articles [23]. This step aimed to fill in any gaps from the two previous strategies and to search beyond the scope of the Acknowledgments field in PMC to find additional mentions of data repositories. It was only possible to perform this search on 10,418 articles for which full-text XML was available via the PMC Open Access Subset [24]; the PMC Open Access Subset includes articles that are still protected by copyright, but are made available via a Creative Commons or similar license that provides for more liberal distribution and reuse of the copyrighted work. This method identified 1,825 articles (**XML dataset**) in total that mentioned a data repository (step 8, Fig 1). We removed these articles from the sample leaving a total number of 69,657 NIH-funded articles that contained “invisible” datasets (Table 6).

**Table 6:** Breakdown for subtraction of articles that mention the deposit of data.

Procedure taken	Articles identified	Articles remaining
1a. NIH-funded articles for 2011 in PubMed	**--**	119,415
1b. NIH-funded articles for 2011 indexed for MEDLINE	6,326	113,089
2. Articles with repository in [SI] field **(SI dataset)**	3,528	109,561
3. Articles with Molecular Sequence Data MeSH Heading **(MSD dataset)**	3,460	106,101
4. PubMed cited articles not available in PMC	24,497	81,604
5. Non-research articles	9,694	71,910
6. Articles with repository in PMC Acknowledgements **(ACK dataset)**	230	71,680
7. Additional articles not available in PMC	198	71,482
8. Articles with repository in full-text XML (of 10,418 searched) **(XML dataset)**	1,825	69,657
Total remaining articles used for subsequent analysis	**--**	**69, 657**

The mention of a dataset or repository in the body of the full-text does not necessarily mean that the data are deposited in the repository; it confirms only the presence of the term(s) in the article, not the context. To estimate the frequency with which a mention of a repository corresponds to the actual deposit of a dataset, we extracted a random subsample of 180 articles from the **XML dataset** (step 9, Fig 1). Two reviewers independently examined each article in the subsample to determine whether or not the data had been deposited in a mentioned repository. The reviewers first examined the context surrounding the mention of the data repository and then, if necessary, the full text of the article. If a determination could not be made by either of these two methods, the reviewers checked the named data repositories for evidence that the data had been deposited. Following the independent reviews, the reviewers met to agree on the final determination for each article.

### Analysis of articles with “invisible” datasets

**Fig 3:**
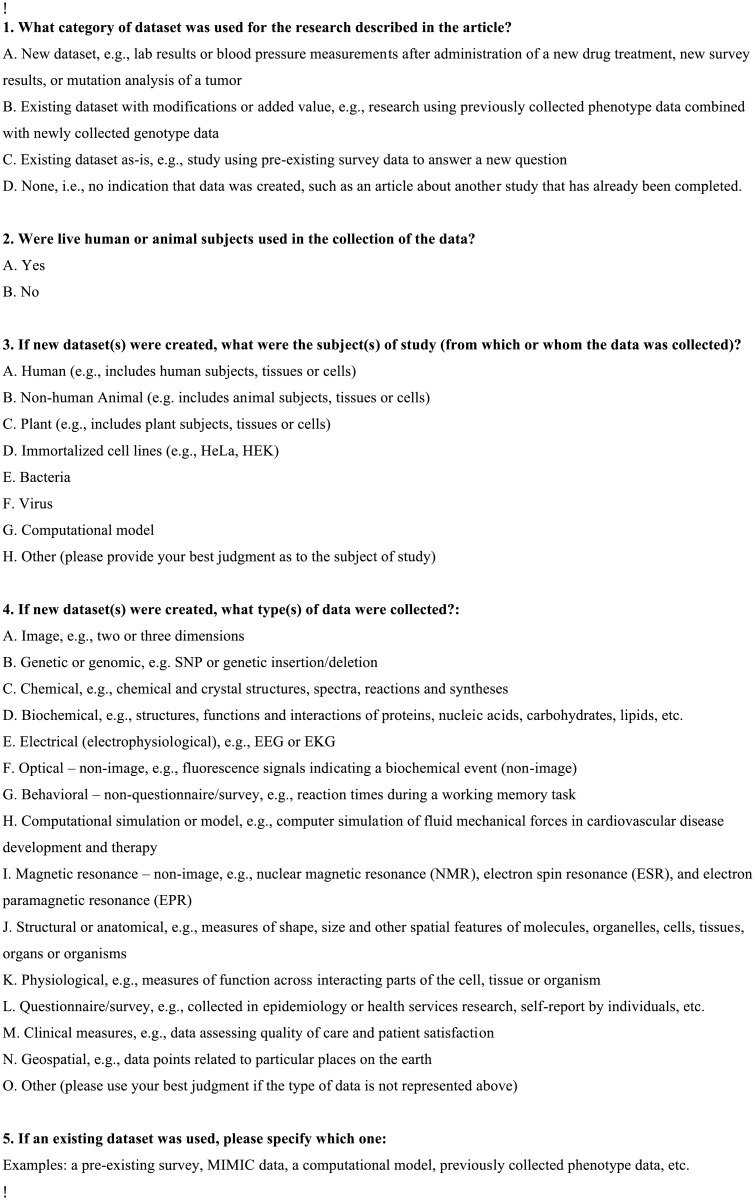
Questions for annotating datasets contained in research articles.

To analyze the 69,657 articles containing “invisible” datasets, we extracted a random sample of 385 articles (confidence interval 95%) for further analysis (step 10, Fig 1). Thirty members of NIH staff were recruited to annotate and analyze the datasets reported in the 385 articles. Annotators were subject experts working in a variety of disciplines including MEDLINE indexers of biomedical literature, biomedical informaticians, physicians, neuroscientists, molecular biologists, librarians, and organizational directors. Each annotator was assigned 25 articles through randomization, and two participants were assigned the same 25 articles—a total of 16 sets—to provide a means to measure the reliability of the counts. One set of annotators only analyzed 10 articles, as this set represented the remaining balance after articles were assigned to other annotators. Each annotator was asked to review his or her assigned articles in their entirety and answer questions related to each dataset described therein. Annotators were instructed to look closely at the methodology section of the paper and any figures or tables to determine how many different measurements were taken. Annotators received a guideline document that included a set of questions to be answered for each assigned article. There was a list of controlled terms for anticipated answers to some of the questions. The guidelines and controlled terms went through several iterations including a pilot study and several internal reviews to improve the clarity of what was being asked and enhance the comparability of the results between annotators. The series of questions are listed in Fig 3.

The categories used to describe the type of data collected in each dataset were developed by the authors of this paper, based on their knowledge of various types of data collected in biomedical research. They do not reflect any particular standard for classifying dataset types. These data types were also informed by an earlier pilot study, and consultation with a variety of stakeholders within the NLM including leadership, indexers, bioinformaticians, and ontologists.

Annotators were asked to populate a spreadsheet with their answers and create a row in the spreadsheet for each dataset found within an article. This procedure provided an opportunity to count how many datasets were created per article, and understand the different types of data that were collected per article. Once annotators completed their 25 articles, the results were returned for review and analysis.

## Results

We first summarize the results of our analysis of datasets in known repositories and then present the results of our analysis of the invisible datasets (Fig 1).

### Datasets in known repositories

#### SI Dataset

**Fig 4:**
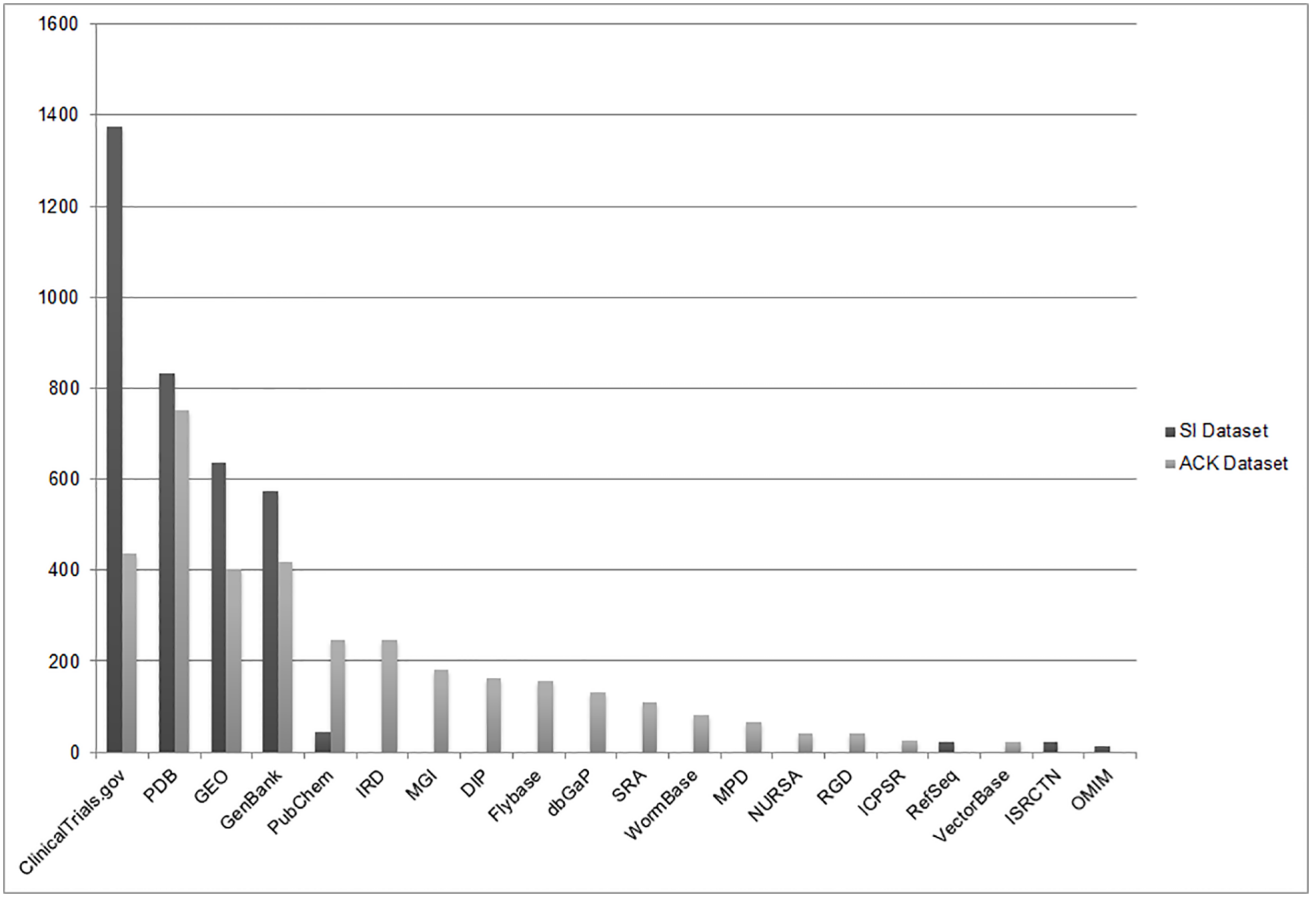
Repositories identified from the PubMed SI field and PMC Acknowledgements where datasets were deposited.

The use of the SI field identified journal articles for which a dataset had been deposited in a specified repository (step 2, Fig 1). It provided valuable information about the common locations from which data are frequently shared. From the original sample of 113,089 MEDLINE citations, more than 3,500 (3.1%) listed data repositories in the **SI dataset**. The most common repositories where data were deposited were ClinicalTrials.gov, Protein Data Bank, Gene Expression Omnibus, and GenBank (Fig 4).

#### ACK Dataset

Review of the PMC Acknowledgements field yielded results similar to the SI field search (step 6, Fig 1). The **ACK dataset** (n = 814) articles acknowledged or mentioned a recognized data repository in more than 3,200 instances, for an average of almost 4 datasets per paper. Protein Data Bank, ClinicalTrials.gov, GenBank, and GEO were again the most common repositories where data were being shared (Fig 4). Because the Acknowledgments search identified a wider range of data repositories than were captured in the SI field in 2011, we were able to gain a better understanding of how often other repositories are used. For example, the Influenza Research Database (IRD), Mouse Genome Informatics (MGI) repository, Database of Interacting Proteins (DIP), and Flybase were the most heavily used data repositories beyond the Protein Data Bank (PDB) and databases managed by NLM. This finding provides insight into the frequency of use of these repositories in a given year (Fig 4).

#### XML Dataset

**Fig 5:**
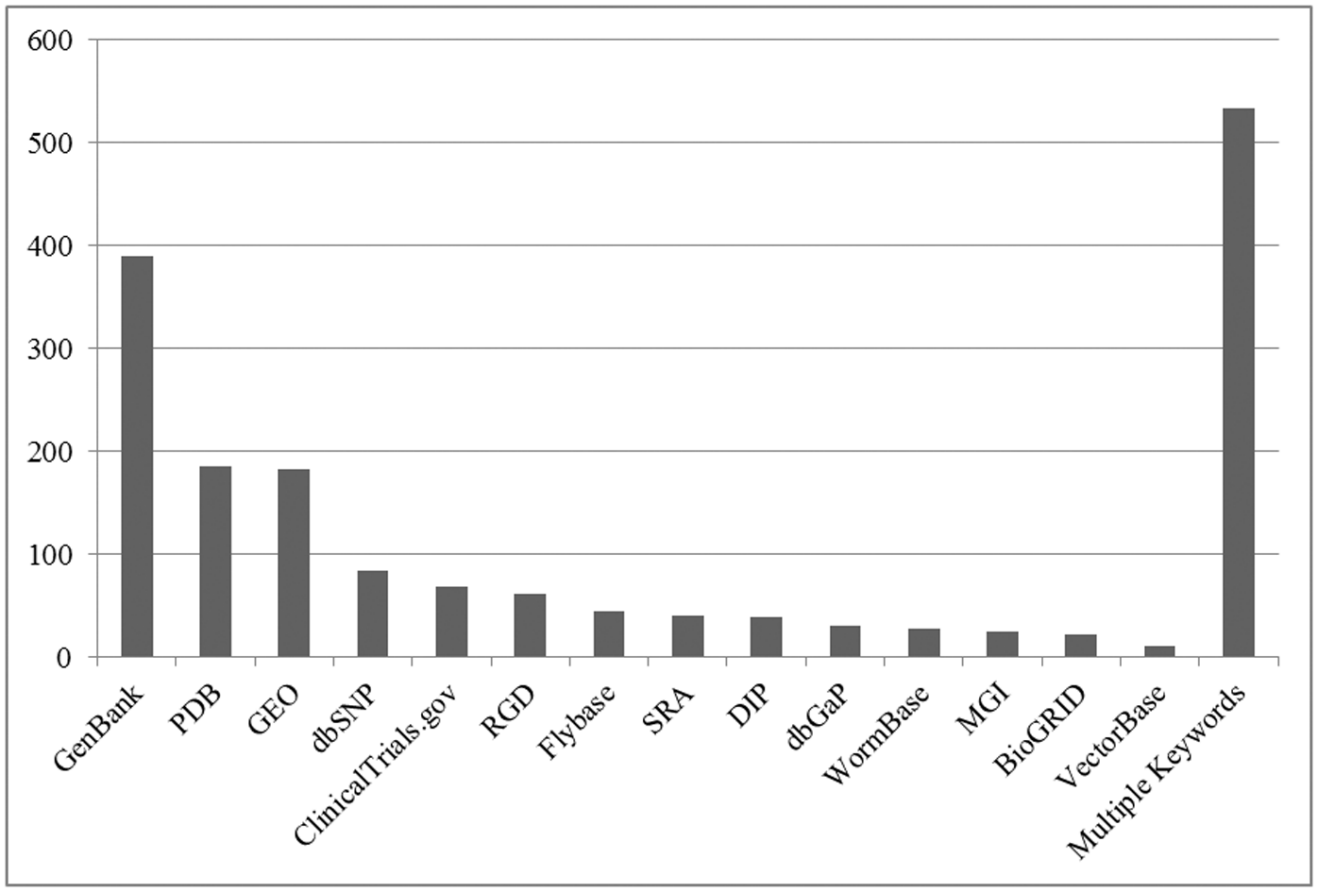
Keywords identified from full-text XML data mining.

The final step to identify journal articles that mention a data repository, the XML method, identified 1,825 additional articles that mentioned a data repository somewhere in the text other than the Acknowledgments section (step 8, Fig 1). As noted in the methodology, this analysis was performed on only 10,418 publications from the PMC Open Access Subset, meaning that 17.5% of the articles analyzed were found to mention a dataset. This finding strongly suggests that any future reviews should be expanded beyond the Acknowledgements section to the entire text of an article. The repositories mentioned in the full-text XML aligned with those identified in the **SI** and **ACK datasets**. GenBank, Protein Data Bank, and Gene Expression Omnibus were again the most prominent data repositories mentioned (Fig 5). This method also identified the long tail of deposits in a number of other specialized repositories. One limitation of this approach is that when multiple data repositories were mentioned in the same article, the XML program could not count the separate repositories individually. This caused all articles that mentioned more than one repository to be categorized together. In total, 29% of all the articles mentioned more than one repository.

In estimating how often the mention of a repository in the XML meant that a dataset had actually been deposited in the mentioned repository, the reviewers determined that 33% of the 180 article subsample taken from the **XML dataset** reflected an actual deposit of data into a data repository (step 9, Fig 1). The remainder fell into four main categories: those that used data from a repository (47%); those that mentioned a repository as background information based on previous research (6%); those that discussed a repository as the subject of the article (4%); or those that used an ambiguous data repository acronym (10%) (e.g., the acronym RGD for Rat Genome Database is also used to describe arginyl-glycyl-aspartic acid). Extrapolating these findings to our larger set, we estimate that 598 articles in the **XML dataset** (33% of 1,825 articles) would refer to the deposit of data into a named repository. This figure is equal to 5.7% of all the articles examined from the PMC Open Access Subset.

The findings from these various analyses provide a rough estimate of the fraction of NIH-funded research articles that indicate that data were deposited in a known public repository (Table 7). From the larger sample of MEDLINE citations that referenced NIH support in 2011, we found that 3.1% had an SI field (**SI dataset**) that indicated deposit in a known repository (step 2, Fig 1). From the remaining set of articles, another 3.2% had molecular data (**MSD dataset**) that would likely be deposited in GenBank or an equivalent repository (step 3, Fig 1). Of the research articles for which full-text was available, 0.3% included a unique acknowledgement of a data repository (**ACK dataset**) that was discoverable by searching using the [ack] search tag in PMC (step 6, Fig 1). Of articles examined from the PMC Open Access Subset (**XML dataset),** an estimated 5.7% reference the deposit of data into a named repository (step 9, Fig 1). Because the percentages are taken from different subsamples of the larger population of NIH-funded articles in 2011, they cannot be strictly summed. As a rough measure, however, they suggest that an estimated 12.3% (Table 7) of the articles published by NIH-funded investigators in 2011 referred to datasets that are, or may be, stored in a known publicly accessible data repository. The datasets in the remaining 88% of published journal articles were “invisible.”

**Table 7:** Estimated Number of Articles with a Dataset Stored in a Known Repository.

Procedure taken	Articles Examined	Articles identified	% of Examined Articles
[SI] field (**SI dataset**)	113,089	3,528	3.1%
Molecular Sequence Data MeSH Heading (**MSD dataset**)	109,561	3,460	3.2%
PMC Acknowledgements (**ACK dataset**)	71,910	230	0.3%
Full-text XML (**XML dataset**)	10,418	598	5.7%
**Estimated Total**	**—**	**—**	**12.3%**

### “Invisible” datasets

Our analysis of the 385 journal articles without a discoverable reference to a data repository is summarized in Table 8 and highlights the challenges in defining and counting datasets. Eight of the 30 annotators defined a dataset as consisting of all of the data resulting from an article, irrespective of the different types of data involved (e.g., chemical test results, imaging data). This meant that half of the sets of articles had only one review that was consistent with our proposed methodology, rather than the two desired. As a result, each set of articles that included only one valid review was removed from the final analysis, leaving 8 sets of articles for analysis (step 11, Fig 1). This reduced sample of 200 articles yielded a confidence interval of 84.3% for our subsequent estimates.

**Table 8:** Summary of Analysis of Invisible Datasets.

Measure	Finding
Average number of datasets per article	All articles reviewed: 3.4 per article
	Articles with two reviews: 2.9 per article
Type of subject	Human subjects: 28.3%
	Non-human animal subjects: 26.1%
New vs. existing data	New datasets: 87%
	Existing datasets: 13%

For these sets of articles with two reviews, there were substantial differences between annotators in the number of datasets identified and described. Within a set of articles, the average difference between the annotators who identified high and low numbers of datasets was 43%—a significantly high percent difference with respect to the validation of this exercise. While the percentage differences are large, however, the absolute numbers are small, and most pairs differed by only one or two datasets. Only one set of annotators counted widely divergent numbers of datasets in their sample.

The analysis nevertheless provides insight into the number of datasets per article. Considering only the eight sets of articles for which there were two valid reviews, the average number of datasets counted per article was 2.9 (Table 8). When all sets of annotations were evaluated (including those sets where annotators counted only one dataset per article), the average number increased to 3.4 datasets per article, reflecting the fact that some of the publications in the additional sets of articles were reported to contain high numbers of datasets. An average of between 2.9 and 3.4 datasets per article aligns with the estimated four datasets per paper we found in our methods for identifying datasets within the **ACK dataset**.

There was greater consistency between pairs of annotators in identifying data from human subjects and live non-human animals. The average percentage of articles identified as reporting research involving human subjects was 28.3%, and the percentage identified as involving non-human animals was 26.1% (Table 8).

The last phase of analysis determined how much of the data that was used in the course of NIH-funded research published in 2011 was new data, and how much was pre-existing data. We counted the percentage of new versus pre-existing data for each set of articles and then calculated the total percentage from all. Annotators were very consistent in making this determination. Combining results from all annotators, we estimate that 87% of the articles involved the collection of new data, and 13% involved the analysis of pre-existing data (Table 8). While new data were collected for purposes of the research reported in the article, pre-existing data included data from previously conducted clinical trials (e.g., reanalysis of the clinical trial data) or surveys (e.g., at local, regional, or national level), among other sources. Some articles made use of both new and pre-existing data.

Annotators were inconsistent in their ability to assign data types from our controlled list of categories to datasets found within articles. Few annotators chose to use the controlled list; most preferred to use the “Other” option to describe the datasets they found, highlighting the difficulty in establishing a suitable classification for biomedical data types (Fig 3).

## Discussion

This study is an initial step toward estimating the additional resources and infrastructure that will be needed to support expanding mandates to make data resulting from NIH-funded and other research available for use by researchers and the public. Methods for discoverability, access, and citation will need to be able to scale in a cost-effective manner if they are to include all such datasets used in published NIH-funded research studies, let alone all datasets used in published biomedical research regardless of funder. It is likely that an increasing number of biomedical datasets will be deposited in general purpose repositories (e.g., Dryad, Figshare), which will affect strategies for enhancing discoverability and access. Data citations are still uncommon and not frequently used by the scientific community. One study found that 88.1% of datasets within the Data Citation Index remained uncited [25]. Another study found that even a national data center rarely could identify formal citations of their data [26]. Without the ability to link and connect research datasets across multiple platforms, discovery and access will remain an issue.

Our results suggest that datasets referenced in only about 12% of articles reporting NIH-funded research in 2011 were (or were eligible to be) deposited in a known, publicly accessible data repository. Our estimates echo findings from previous studies that indicate a large portion of datasets are not shared. In one study, only 9% of articles from high-impact journals deposited their full dataset (including raw data) online [12]. Another study found that in their sample not a single study cited a dataset with a unique identifier, therefore providing no indication that the data are shared anywhere [13]. Our analysis also found that an expected 858 articles (47% of 1,825 articles) in the **XML dataset** mention the use of data from a known repository (rather than a deposit into a repository). This figure is equal to 8.2% of the articles we examined from the PMC Open Access Subset and provides a measure of the level of reuse of data from known repositories.

Our findings also help characterize the invisible datasets from biomedical research that are not deposited in a known repository. We found 69,657 articles that were published in 2011 and reported on NIH-funded research but did not indicate that data had been deposited in a known repository. We estimate that the research described in these articles used an average of 2.9 to 3.4 datasets per article. These figures mean that approximately 200,000 to 235,000 datasets were used in NIH-funded research published in 2011 but not deposited in one of the well-known public repositories for specific categories of biomedical data (e.g., GEO, GenBank, Protein DataBank, ClinicalTrials.gov).

Given that as many as 88% of biomedical research datasets may not currently be deposited in a well-known, public data repository, the problem of improving the discoverability of biomedical datasets remains significant. The basic challenge is therefore deciding which data are most worthy of the additional resources and effort that will inevitably be required to make them readily discoverable and accessible. Arguably, useful and manageable data discovery systems should focus on datasets that show potential for reuse or that point to significant findings so that underlying data should be available to others to assess validity and accuracy. A strong case can be made that datasets derived from live subjects have a particularly high priority for discoverability and accessibility. Making such datasets readily available could reduce the need to expose additional live subjects to potential risks and, in the case of human subjects, help to meet the ethical obligation to ensure that their participation in research studies adds to scientific knowledge.

Another point to consider is *how* data can be best discovered, accessed, and understood. Our review of datasets suggests that while some datasets (such as those stored in known repositories like ClinicalTrials.gov, GenBank, and Protein DataBank) can stand on their own and serve as resources for other investigators, many datasets may have limited utility outside the study for which they were collected. These datasets may be meaningful only when considered alongside other datasets collected for the same study and in conjunction with the journal article that summarizes them. This observation has significant implications for data discovery and storage, because it suggests that in some cases the preferred discovery tool may be the publication where the datasets are described rather than a separate mechanism that would find and retrieve them individually and independently. This argument has already been addressed within the scientific community, with some calling for an advanced publication where the underlying data can be extracted directly from the paper [27–30]. Nanopublications are another development that shed light on providing context for datasets pulled from a scientific paper; these abridged data publications provide narrative descriptions of data pulled directly from an article [31]. It is our belief that including dataset metadata summaries within the published article may be an efficient way to promote the discovery of these datasets.

Further work is also needed to determine how to define a dataset. As evidenced by the lack of consistency between annotators with respect to the number of datasets they identified, there are differences in perceptions of what constitutes a dataset and in how well data collected or used in a research study are described in journal articles. Depending on one’s perspective, a single dataset could be: all of the data that is collected or used in a study; all data collected at a specific time within a study; pre- or post-intervention; a discrete type of data from a specific diagnostic device; or even every individual measurement reported in a research article. Data access and sharing requirements must clearly define a dataset to outline expectations of what researchers will be required to share and submit and what will be available to potential users. Requirements are likely to vary depending on the use (or reuse) cases for different types of data.

Data creation and analysis pipelines raise additional questions about how data should be described and at what point along the pipeline. Collected data go through multiple processing transformations during analysis. As a simple example, an image may be collected of a cell (e.g., an optical image); that image may be analyzed by measuring the size of certain structures in the cell (e.g., numerical data); the numerical/structural data from multiple cells may be aggregated for analysis (e.g., to compare the size of structures in treated versus untreated cells). Results of that analysis may be shown in a table or a graph, perhaps showing trends in size of the structures in the treated and untreated cells over time. For a researcher interested in reproducing the research, the basic imaging data may be of most interest, but such a researcher might also want or need to know how the data were reduced and need access to associated data processing algorithms. For a researcher interested in comparing results across studies, the more processed data may be of most interest. For a researcher interested in reusing the data, the data from a particular point along the data processing pipeline might be most useful. Providing data at each step along the pipeline might prove to be onerous or overly complex for data generators and those who want to make use of the data.

Any system for data discovery and access must describe data in a way that will be useful for those researchers, health professionals or members of the public who are interested in reviewing biomedical data. An examination of a variety of metadata schemas [32–34] and the metadata employed in existing NIH data repositories indicates that the baseline description of datasets in current repositories does not differ greatly from descriptive metadata for journal articles or archival objects. However, we are not aware of strong evidence that the current metadata schemes applied to biomedical datasets either do or do not meet the needs of researchers seeking data to reuse. There has been considerable discussion about including enriched metadata to make data more discoverable in the context of a data publication to provide detailed metadata and description of individual datasets [35, 36]. Some research has begun to examine the quality of metadata used in scientific data repositories [37], but more research is needed to determine what metadata would enable efficient discovery of various types of data. Analysis of current use patterns of existing repositories that accommodate disparate datasets may shed light on what types of data and descriptive metadata are most useful.

Determining which types of biomedical data have the highest reuse value, how to describe them usefully and cost-effectively, and how to make them accessible in a sustainable way are key challenges for the NIH and its recently established Data Discovery Index Coordination Consortium [38] as they move forward to make biomedical big data more discoverable, accessible, and citable.

## Conclusion

These findings represent a first look into the landscape of NIH-funded data. An understanding of the varying types of data that are created throughout the course of biomedical research and the knowledge that a substantial amount of new data is created per article in a given year will help to inform efforts to improve the discoverability and accessibility of digital biomedical research data. Differences in perspective encountered among participants in the study suggest that the creation of data discovery tools for biomedical research data will not be straightforward. Decisions will have to be made as to what data will be selected for description and careful consideration will need to be given to identifying how to describe datasets derived from NIH-funded research.
